# Magic Cures II

**Published:** 1893-01-28

**Authors:** 


### MAGIC CURES.—II.

It has been seen that in the great majority of cures attributed to occult causes, the main factor was either the doctrine of a vicarious sacrifice, a life for a life, or the belief in a wholly mysterious sympathy between the powers of nature and man, producing far-reaching results from the most apparently idle or grotesque actions.

Some minor points in these cures, recurring with great persistency in the recorded accounts of them, remain yet to be noticed. In the description already quoted of the recovery of the infant Pascal, it will be remembered that a great feature of the cure was the poultice made of nine leaves of three kinds of herbs, three of each sort. The nature of the herbs seems to have been a matter of indifference; the virtue lay in the three times three. As old as Pythagoras, this faith in the mystic properties of numbers has never failed mankind. It still retains its hold, not only on the gambler and speculator in lotteries, but even on the mild and respectable persons who frequent bazaars and indulge in the modest excitement of a raffle. And while the old-fashioned mixture is still reluctantly swallowed three times a day, and the homœopath sips thrice daily his little drink prepared in nine teaspoonsful of water, can we say that modern medicine retains no trace of the old leaven?

The curious thing is that almost every number has held a sacred character assigned to it at one time or another, so that it might seem a matter of indifference which was selected. Nine, as containing the square of three, stands, perhaps, at the head of the list; seven is the next favourite, and these two numbers multiplied into each other gave the grand climacterical year, viz., 63—an age deemed by the ancients one of peculiar danger to be guarded by every medical precaution available. The great advocate for five is Sir Thomas Browne in his "Quincunx," and the same author remarks that "six hath found many leaves in its favour." The Pythagoreans swore by the number four, while ten "hath been highly extolled as containing even, odd, long, plain, quadrate, and cubical numbers." As a general principle odd numbers are preferred to even, "wherefore it is that many doctors prescribe always an odd pill, an odd drop or draught to be taken by their patients." It must not be forgotten in this connection that "the seventh son of a seventh son is born a physician, having an intuitive knowledge of the art of curing all disorders, and sometimes the faculty of performing wonderful cures by touching only."

Among instances of the place of number in magic cures may be cited one for jaundice, quoted as wonderfully efficacious by Dr. Edward Browne in his journal. The instrument of cure was wood ashes prepared in a peculiar manner, and the method concludes: "Then lay nine long heaps of the boyld ashes upon a board in a ranke, and upon every heap lay nine spears of crocus; it hath greater effects than is credible to anyone that shall barely read this receipt without experiencing."

In the "Gentleman's Magazine" for 1794, a silver ring is mentioned as a cure for fits, "made of five sixpences, collected from five different bachelors, to be conveyed by the hand of a bachelor to a smith that is a bachelor." But instances of this application of a magic number to disease will readily occur to every reader.

The element of colour, conspicuous in the history of magic, held an important place also in mediæval medicine. In diseases of the blood, for instance, red flowers were prescribed, while yellow drugs were in esteem for complaints of the liver. The red treatment for small-pox was much in vogue at one time, and seems to have been beneficial in preserving the patient from more dangerous attempts at cure. John of Gaddesden, physician to Edward II., boasts not unnaturally of the success of this treatment. He says: "When the son of the renowned King of England lay sick of small-pox, I took care that everything around the bed should be of a red colour; which succeeded so completely that the prince was restored to perfect health, without a vestige of a pustule remaining."

Mr. Pettigrew asserts, moreover, that flannel dyed nine times in blue was held to be efficacious in removing glandular swellings.

The belief in the influence of the stars was answerable for all the curious regulations as to the due time for gathering herbs used in mystic cures. Burton, for example, in asserting that St. John's wort "borne or hung about the neck will mightily help melancholy and drive away fantastical spirits," warns the patient that the plant must be gathered on Friday, in the horn of Jupiter, when it comes to his effectual operation, that is, about the full moon in July. Not only was a special virtue attached to certain times of the day and year, but so many days were marked off as unpropitious to the taking of drugs that Sir Thomas Browne complains that the ancients have left only a scanty quarter of the year in which the physician could lawfully exercise his art. Yet, doubtless, some unhappy sufferers in those days may have deemed this more than enough.

Lastly, in the cure of disease an extraordinary efficacy was held to attach to certain places. Cross roads were held in mingled esteem and horror; esteem, because the sign of the cross was held to scare away malicious spirits; horror, because on this account often selected as the burying-place of the suicide.

The special sanctity which the Romanists attach to certain spots often originated in the desire to Christianise some old pagan observance; thus, where it had been usual to invoke the name of a demon, that of a saint might be more properly substituted. Such spots were often wells or springs, and a curious mixture of observances, religious and magical, betray in many instances their double origin. The holy well, Tubber Quan, near Carrick-on-Suir, affords a good illustration of these dual rites. "The well is dedicated to two patron saints, St. Quan and St. Brogawn. The times for visiting it are the last three Sundays in June. It is firmly believed that at this period the two saints appear in the well in the shape of two small fishes, of the trout kind, and if they do not so appear that no cures will take place. The penitents attending on these occasions ascend the hill barefoot, kneel by the stream, and repeat a number of paters and aves, then enter it, and go through the stream three times at a slow pace, reciting their prayers. They then go on the gravel walk, and traverse it round three times on their bare knees, often till the blood starts in the operation, repeat their prayers, then traverse three times round a tree on their bare knees, but upon the grass. Having performed these exercises, they cut off locks of their hair and tie them on the branches of the trees as specifics against headache. The tree is a great object of veneration, and presents a curious spectacle, being covered all over with human hair." Note that almost every point to which we have called attention in these magical cures is here present—the blood, a substitute for a life, the transference of pain by sympathy to an inanimate object, the element of number (and, as usual, three times three), the mystic season, and the sanctity of place.

A very forcible example of the revival, under scientific forms, of old superstitions is afforded by the extraordinary reputation in our own day of many of these old springs. Many of the causes which contributed to bring about well-attested cures, such as vigorous exercise, early rising, abstemious living, and exposure to sun and air (not to mention the mineral properties of the water and the advantages of cleanliness), are now rightly assigned, and hence the consulting physician, in touch with the latest developments of modern science, is often found treading unconsciously in the steps of the mediæval saltimbanco, prescribing for his wealthy city patient precisely the same course of unusual exertion and foreign travel which would have been marked out for him by superstition four or five hundred years ago.

